# Anticarcinogenic potentials of tea catechins

**DOI:** 10.3389/fnut.2022.1060783

**Published:** 2022-12-05

**Authors:** Xiao-Xiang Li, Chang Liu, Shu-Ling Dong, Can-Song Ou, Jian-Liang Lu, Jian-Hui Ye, Yue-Rong Liang, Xin-Qiang Zheng

**Affiliations:** ^1^Tea Research Institute, Zhejiang University, Hangzhou, China; ^2^Tea Science Society of China, Hangzhou, China; ^3^Development Center of Liubao Tea Industry, Cangwu, China

**Keywords:** *Camellia sinensis*, tea catechins, anticancer, antioxidant, free radicals, synergistic interaction, metastasis, signaling pathway

## Abstract

Catechins are a cluster of polyphenolic bioactive components in green tea. Anticarcinogenic effects of tea catechins have been reported since the 1980s, but it has been controversial. The present paper reviews the advances in studies on the anticarcinogenic activities of tea and catechins, including epidemiological evidence and anticarcinogenic mechanism. Tea catechins showed antagonistic effects on many cancers, such as gynecological cancers, digestive tract cancers, incident glioma, liver and gallbladder cancers, lung cancer, etc. The mechanism underlying the anticarcinogenic effects of catechins involves in inhibiting the proliferation and growth of cancer cells, scavenging free radicals, suppressing metastasis of cancer cells, improving immunity, interacting with other anticancer drugs, and regulating signaling pathways. The inconsistent results and their causes are also discussed in this paper.

## Introduction

Cancer or carcinoma is a leading contributor to the global disease burden and the number of its deaths is next only to cardiovascular diseases (1). There were 23.6 million cases of various cancers [95% confidence interval (95% CI) = 22.2∼24.9 million] and 10.0 million deaths (95% CI = 9.36∼10.6 million) in 2019 across 204 countries and territories (1). Projections forecasted that the worldwide cancer burden might continue to increase for the next two decades (2–4) and it is supposed to have 28.4 million cancer cases in 2040 (5). It is considered that diet will be an effective pathway for preventing some cancers (6, 7).

Catechins are a cluster of bioactive polyphenolic components in fresh tea leaves or green tea and they are major contributors to the health benefits of tea (6, 8). There are usually four epi-type catechins found in fresh leaves of normal tea cultivars, i.e., (−)-epicatechin (EC), (−)-epigallocatechin (EGC), (−)-epicatechin gallate (ECG), and (−)-epigallocatechin gallate (EGCG). Their isomers, however, are detected in made teas due to heat-induced epimerization from the epi-type catechins in tea manufacture (9, 10). The most abundant individual of catechins in fresh tea leaves is EGCG which is more than 40% of the total content of catechins (8). Many studies showed the anticarcinogenic effects of tea catechins (11–13). However, the effects of tea catechins and tea consumption on the risk of cancers is still inconclusive and controversial (14, 15). Though there were dozens of review papers focusing on this topic in the last decade, the controversial or inconsistent results, which are very important for the future studies, have not been involved. This review not only summarizes the progresses on the anticarcinogenic effects of catechins or tea and underlying mechanism, but also highlights the controversial or inconsistent results and their causes, which will be provide helpful references for future studies.

Original research papers or epidemiological study reports were researched on the Web of Science using key words “catechins (topic) and anticarcinogenic (topic) or anticancer (topic)” and those with no relation to catechins from tea source were excluded.

## Epidemiological evidence of anticarcinogenic effects of tea and tea catechins

There have been epidemiological studies showing the relationship of tea consumption to the decreased risk of cancer incidences since the end of the last century. A 9-year study among 8,552 Japanese adults revealed that frequent consumption of green tea in large amounts showed a potentially beneficial effect on cancer prevention. The cancer onset was delayed by 8 years in females, but by 3 years in males who consumed ≥10 cups of green tea daily, compared to those who consumed ≤3 cups daily (16). Meta-analysis including 18 prospective cohort and 25 case-control studies showed a significant inverse association between intake of tea catechins and risk of various cancers, with a relative risk (RR) being 0.935 (95% CI = 0.891∼0.981). Catechins consumption showed significantly protective effect on breast cancer (BC) (RR = 0.885, 95% CI = 0.790∼0.991), rectal cancer (RR = 0.838, 95% CI = 0.733∼0.958), oropharyngeal and laryngeal cancer (RR = 0.759, 95% CI = 0.581∼0.993), and stomach cancer (RR = 0.633, 95% CI = 0.468∼0.858) (17).

### Gynecological cancers

Tea drinking is found to be related to the reduced risk of gynecological cancers. A case-control study on Asian-American women in Los Angeles, USA revealed that green tea intake was significantly and negatively correlated to the BC risk (18). Furthermore, a study involving 472 patients with stage I, II, and III BC in Japan showed that green tea consumption was negatively correlated to numbers of axillary lymph node metastases among pre-menopausal patients with stage I and II BC, but positively correlated to the expression of estrogen receptor (ER) and progesterone receptor (PgR) among post-menopausal patients. A 7-year follow-up study on these patients showed that an increase in green tea consumption was negatively correlated to the recurrence of stage I and II BC (*p* < 0.05), with a recurrence rate being 16.7% among patients who consumed ≥5 cups/day, but being 24.3% among those who consumed ≤4 cups/day. After adjustment for other lifestyle factors, the RR of recurrence among patients with stage I and II BC was 0.564 (95% CI = 0.350∼0.911) (19). In a population-based case-control study involving in 501 BC patients and 594 control subjects among Japanese, Chinese, and Filipino-American women, which was carried out during 1995–1998, showed that the risk of BC decreased with increase in intake of green tea [odds ratio (OR) = 1.00, 95% CI = 0.71 (0.51∼0.99)] in non-tea drinkers (0–85.7 mL/d) and OR = 0.53 (95% CI = 0.35∼0.78) in tea drinkers (>85.7 mL/d) (20). A meta-analysis based on data through literature search in the MEDLINE database from January 1966 to August 2004, which revealed the relationship of black tea or green tea consumption to BC risk among populations from eight countries, showed that high intake of green tea reduced the risk of BC with OR = 0.78 (95% CI = 0.61∼0.98). Black tea consumption showed weakly positive correlation to the risk of BC, with OR = 0.91 (95% CI = 0.84∼0.98), in which the negative correlation was higher in hospital-based studies (OR = 0.77, 95% CI = 0.50∼1.19) than the population-based case-control studies (OR = 0.94, 95% CI = 0.81∼1.09) (21). An investigation on 3,315 women in Singapore showed that mammographic density (PMD) of daily green tea drinkers (19.5%) was significantly lower than that of non-tea drinkers (21.7%, *p* = 0.002) after the relevant covariates were adjusted, however, the intake of black tea was not associated with the risk of PMD (22). A meta-analysis including 3,323,288 participants showed that heavy tea consumption has a preventing effect against ER-BC, especially in the post-menopausal women. Therefore, tea was considered to be a potentially useful dietary protectant for preventing BC and a recommended dose was ≥5 cups/day (23).

Ovarian cancer risk was found to be associated with tea drinking. Black tea consumption showed linearly suppressive effects on the risk of ovarian cancer (*p* = 0.03), in which 30% decline in the risk was observed (adjusted OR = 0.70, 95% CI = 0.51∼0.97) among those who consumed ≥2 cups daily (24). Green tea drinking was found significantly to decrease the risk of ovarian tumors (OR = 0.81, 95% CI = 0.73∼0.89, *p* < 0.0001) in a meta-analysis study including 3,842 cases and 5,271 control cases retrieved from Wanfang, CNKI, CBMdisc, EMbase, and PubMed databases during 2000–2010 (25).

Tea drinking was found to be beneficial to endometrial cancer. A meta-analysis involving 6,797 cases of endometrial cancer and 858,780 normal controls showed that the pooled RR of endometrial cancer for the highest consumption of tea was 0.99 (95% CI = 0.94∼1.04, *p* = 0.005), compared with the lowest level. Further study showed that the pooled RR was 0.83 (95% CI = 0.73∼0.95) for case-control study but 1.02 (95% CI = 0.96∼1.08) for cohort study, in terms of study design. For various regions, however, the pooled RR was 0.80 (95% CI = 0.69∼0.93) for Asia, 1.06 (95% CI = 0.94∼1.20) for USA/Canada and 1.01 (95% CI = 0.95∼1.08) for Europe. For different kinds of tea, inverse associations of reduced risk of endometrial cancer were found with black tea (RR = 0.65, 95% CI = 0.46∼0.92) and green tea (RR = 0.73, 95% CI = 0.64∼0.84), respectively (26).

### Digestive tract cancers

Epidemiological investigations showed that tea drinking was correlated with a reduced risk of digestive tract cancers. An 8-year follow-up cohort study on association between tea consumption and cancer incidence involving 35,369 post-menopausal women in Iowa, USA showed that increasing frequency of tea drinking was inversely associated with digestive tract cancer (*p* = 0.04, RR = 0.68, 95% CI = 0.47∼0.98) for females who drank ≥2 cups (474 mL/cup) daily, in comparison with the women who occasionally or never drank tea (27). A population-based case-control study including 931 colon cancer, 884 rectum cancer, 451 pancreas cancer patients, and 1,552 control residents which was conducted in Shanghai, China during 1990–1993, showed that the amount of green tea consumption was inversely associated with colorectal and pancreatic cancers (28). Green tea drinking reduced by 39, 78, and 81% in the risk of esophageal cancer, liver cancer and gastric cancer were, respectively observed among alcohol drinkers according to a population-based case-control study conducted in Taixing, China (29). Those with a high level of pre-diagnostic urinary epigallocatechin (EGC) (a component of tea catechins) had a lower risk of colon cancer. The ORs for colon cancer in populations with the lowest, intermediate, and highest tertile of EGC detected were 0.64 (95% CI = 0.33∼1.24), 0.60 (95% CI = 0.30∼1.20), and 0.40 (95% CI = 0.19∼0.83) respectively (*p* < 0.02) in comparison to those without EGC detected. Methylated EGC showed a similar effect on colon cancer (30). A 6-year population-based prospective cohort study involving 14,001 elderly inhabitants with ages ranging from 65 to 84 years in Shizuoka, Japan showed that green tea drinking was negatively correlated with colorectal cancer (CRC) mortality in a mediate dose dependent manner, with hazard ratio (HR) = 0.24 (95% CI = 0.14∼0.40) for total participants, HR = 0.30 (95% CI = 0.15∼0.61) for men, and HR = 0.18 (95% CI = 0.08∼0.40) for women who consumed ≥7 cups/day, compared to those who drank more than one cup daily (31). A pooled analysis of six cohort studies including 219,080 subjects, among which 3,577 cases were gastric cancer, showed a significant risk reduction for gastric cancer for women with consumption of more than 5 cups daily (multivariate-adjusted pooled HR = 0.79, 95% CI = 0.65∼0.96), but no significant risk reduction for gastric cancer in men (32).

Green tea intake was shown to be negatively correlated with stomach cancer risk (RR/OR = 0.86, 95% CI = 0.74∼1.00). The group with green tea consumption >5 cups/day showed significantly preventive effect on stomach cancer (RR/OR = 0.68, 95% CI = 0.53∼0.87) (33). An 11-year follow-up study involving 1,255 persons suffering from digestive system cancers including esophagus, stomach, liver, pancreas, colorectal and gallbladder/bile duct cancers from 69,310 non-alcohol-drinking and non-smoking women in Shanghai, China showed that normal tea drinking (more than three times per week for more than 6 months) was showed suppressive effects on the overall digestive system cancers (HR = 0.86, 95% CI = 0.74∼0.98, *p* = 0.01). In comparison with women who never drank tea, and the risk decreased with increase in duration and amount of tea drinking (*p* < 0.01) (34). A cohort study involving a total of 65,042 Japanese residents aging from 40 to 79 years revealed that green tea consumption showed a protective effect against hematologic neoplasms, with multivariate HR = 0.65 (95% CI = 0.42∼1.00) for leukemia among those who consumed 2 cups of green tea daily or less, 0.73 (95% CI = 0.47∼1.13) for those who drank 3–4 cups of green tea daily, and 0.63 (95% CI = 0.42∼0.96) for those who drank 5 cups of green tea daily (35). An investigation involving 7,355 eligible subjects who were classified into polyp-free, low-risk colorectal adenomas and high-risk colorectal adenomas based on health check-ups with colonoscopies showed that tea drinking was inversely associated with low-risk colorectal adenomas. A larger cumulative dose (≥42 cup-year) was negatively associated with high-risk colorectal adenomas, especially adenomas with villous-rich pathology and when three or more adenomas were present (36).

### Incident glioma

Tea consumption was revealed to be negatively correlated to the risk of glioblastoma (HR = 0.93, 95% CI = 0.89∼0.98) in a prospective cohort study involving 379,259 UK Biobank participants among which 487 were incident glioma cases. Consuming ≥4 cups/day was decreased the risk of glioma in comparison of no tea consumption (HR = 0.69, 95% CI = 0.51∼0.94) (37). A study on relationship of recent (up to 12 years) or average long-term (up to 30 years) dietary flavonoid intake to the risks of incident glioma based on data from the male Health Professionals Follow-Up Study (1986–2014, *n* = 49,885), Nurses’ Health Study II (1991–2017, *n* = 95,228) and the female Nurses’ Health Study (1984–2014, *n* = 81,688) showed that long-term tea catechins intake was associated with reduced risks of glioma in pooled analysis with comparison of the highest quintile to the lowest quintile of tea consumption (HR = 0.76, 95% CI = 0.57∼1.01, *p* = 0.04). The association with recent intake was weaker (38). Meta-regression analysis showed that higher tea consumption was related to a reduced glioma risk (RR = 0.84, 95% CI = 0.71∼0.98, *p* = 0.030), and sensitivity study through removing case-control studies revealed that more consumption of tea was associated with lower risk of glioma (RR = 0.81, 95% CI = 0.70∼0.93, *p* = 0.004). Daily 1 cup of tea reduced the glioma risk by 3% (RR = 0.97, 95% CI = 0.94∼1.00, *p* = 0.048) (39).

### Liver and gallbladder cancers

A population-based case-control study including 1,037 cases with biliary stones, 627 incident cases with biliary tract cancer (BTC) and 959 randomly selected controls of biliary tract disease in Shanghai, China, showed that female tea drinkers have significantly decreased gallbladder cancer risk (OR = 0.56, 95% CI = 0.38∼0.83) and biliary stone risks (OR = 0.73, 95% CI = 0.54∼0.98). However, these risk assessments among male tea drinkers who were more likely to be cigarette smokers were not significantly lower than the non-tea drinkers (40). A 9-year follow-up cohort study involving in 41,761 Ohsaki residents with ages from 40- to 79-year old in Japan showed that green tea drinking was negatively correlated to the liver cancer risk. The multivariate-adjusted HRs for the risk of liver cancer among women were 0.68 (95% CI = 0.35∼1.31) for those who drank 2 cups or less daily, 0.79 (95% CI = 0.44∼1.44) for those who drank 3–4 cups daily, and 0.50 (95% CI = 0.27∼0.90) for those who drank >5 cups daily, compared with the group drinking less than 1 cup/day. The corresponding HRs among men were 0.83 (95% CI = 0.53∼1.30) for 1–2 cups/day, 1.11 (95% CI = 0.73∼1.68) for 3–4 cups/day, and 0.63 (95% CI = 0.41∼0.98) for >5 cups/day (41). Studies on tea consumption in relation to primary liver cancer from 1979 to 2009 in China, which included 13 epidemiological investigations consisting of seven prospective cohort and six case-control studies, showed that consumption of polyphenols abundant tea was negatively correlated with the risk of primary hepatic cell carcinoma (HCC) (RR = 0.77, 95% CI = 0.57∼1.03). Tea drinking showed preventive effects on the development of primary liver HCC both among women (RR = 0.54, 95% CI = 0.37∼0.79) and men (RR = 0.86, 95% CI = 0.77∼0.95) (42). Larger quantities and longer duration of green tea drinking were negatively correlated with primary HCC, among which individuals who consumed green tea more than 30 years showed the lowest risk (adjusted OR = 0.44, 95% CI = 0.19∼0.96) in comparison with non-tea drinkers (43).

### Lung cancer

Heavy drinking of tea (more than 2 cups per day) was found to be negatively correlated with the risk of lung cancer in a case-control study including 428 hospitalized controls and 427 lung cancer cases in Uruguay (RR = 0.34, 95% CI = 0.14∼0.84) (44). Green tea drinking was found to have a reduced primary lung cancer risk in a case-control study including 649 female patients with primary lung cancer based on the data from Shanghai Cancer Registry from February 1992 to January 1994. However, the effect was dependent on smoking. The risks reduced with the increase in green tea consumption among non-smoking women (OR = 0.65, 95% CI = 0.45∼0.93), but little association was found among female smokers (OR = 0.94, 95% CI = 0.40∼2.22) (45). An investigation on the relationship of diet and physical activity to the risk of lung cancer in the Czechia revealed that black tea drinking had preventive effect on lung cancer among non-smokers (OR = 0.67, 95% CI = 0.46∼0.99) (46). The frequency of black tea drinking (daily or several times per week) showed a reduced risk of lung cancer female non-smokers (OR = 0.65, 95% CI = 0.43∼0.99) (47).

### Other cancers

Consumption of hot black tea was found to significantly reduce skin squamous cell carcinoma (SCC) risk (OR = 0.22; 95% CI = 0.10∼0.51) in a population-based case-control study conducted by the Southeastern Arizona Health Study (SEAHS) from 1994 to 1996 (48). Prostate cancer risk was significantly decreased with an increase in quantity, duration and frequency of green tea drinking in a case-control study including 130 incidental prostate cancer cases confirmed histologically and 274 cases without cancer matched by age in southeast China (49). A study in Italy revealed that orally administrating green tea catechins (600 mg/day for 1 year) showed cancer chemoprevention effect on prostate cancer among patients who were at high risk to develop prostate cancer and had pre-malignant lesions (50). A study based on 3 nutrient-specific databases developed by the United States Department of Agriculture which included 466 cases of non-Hodgkin lymphoma (NHL) and 390 controls showed that higher intakes of green tea components including epicatechin, flavonols, proanthocyanidins and anthocyanidins significantly reduced NHL risk (51).

It was found in a case-control study including 107 leukemia cases and 110 orthopedic controls in China mainland that the risk of leukemia decreased with the increase in frequency, quantity and duration of green tea consumption (52). An investigation including 252 cases and 637 controls in China Taiwan revealed that green tea consumption showed significantly negative correlation with leukemia risk among persons from 16- to 29-year-old (OR = 0.49, 95% CI = 0.27∼0.91). The adjusted OR for heavy tea drinking group (more than 550 cups annually) was 0.47 (95% CI = 0.23∼0.97), compared with non-tea drinking group. Large amounts of tea catechins intake showed a reduced risk of leukemia (OR = 0.49, 95% CI = 0.27∼0.91) after adjusting for smoking status and medical irradiation exposure (53). A prospective UK Biobank cohort study involving more than 500,000 participants with ages ranging from 38 to 73 years at enrollment (2006–2010) revealed that tea drinking showed significantly negative correlation to cancer mortality in both male and female (54).

## Anticarcinogenic mechanism of tea catechins

### Inhibition of cancer cell proliferation and growth

Major components of green tea polyphenols (GTPs) are catechins which include at least eight compounds, i.e., EC, ECG, EGC, EGCG, (+)-catechin-3-gallate (CG), (+)-gallocatechin-3-gallate (GCG), (+)-gallocatechin (GC), and (+)-catechin (GC). The content of EGCG accounts for more than 40% of total content of catechins and so it is the most abundant one (8–10). The research on anticarcinogenic activity of catechins has started since the 1980s (55). EGCG showed a dose-dependent inhibitory effect on human papillomavirus type 16 (HPV-16) induced cervical cancer cell CaSki, in which the inhibitory dose (ID) was 35 μM approximately. Cell cycles were arrested at the G1 phase when incubated with 35 μM EGCG and EGCG-induced apoptosis was occurred after incubation at 100 μM EGCG for 24 h (56). When non-small cell lung cancer A549 cells were incubated with catechin at 600 μmol/L for 24 h, inhibition rate of the cell proliferation was 19.76% (57). The inhibitory effects of catechins on proliferation of cancer cells depend on the components of catechins and the types of cancer cells. For the HSC-2 carcinoma cells, ECG, CG, and EGCG were grouped as highly toxic, EGC as moderately toxic, and C and EC as least toxic. For the HGF-2 fibroblasts, ECG and CG were grouped as highly toxic, EGCG as moderately toxic, and EGC, C, and EC as least toxic (58).

**FIGURE 1:**
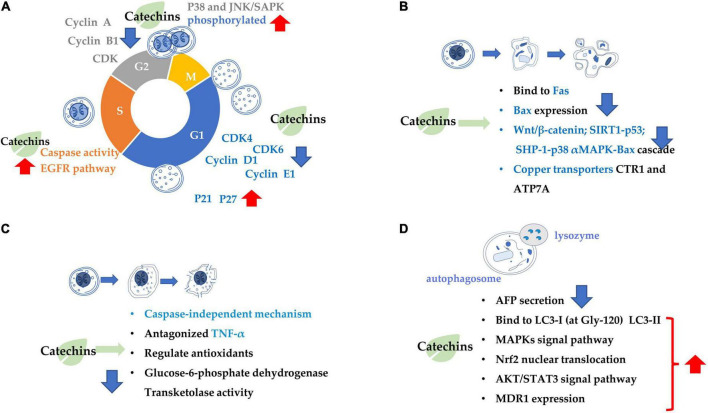
Summary of the target of tea catechins inhibiting cancer cell proliferation and growth. This is mainly achieved through four processes: **(A)** cell cycle arrest; **(B)** cell apoptosis; **(C)** cell necrosis; and **(D)** cell autophagy. Blue arrows indicate downregulation and red arrows indicate upregulation.

The mechanism inhibiting cancer cell proliferation involves in several aspects, such as cancer cell growth arrest and cancer cell death (Figure 1). Catechin inhibits A549 cells by regulating its cell cycle arrest, increasing the expressions of p21 and p27 and inhibiting the expressions of p-AKT (phosphorylated protein kinase B) and cyclin E1 in a dose-dependent manner in the cancer cells, which also contributed to the suppression of cancer cell proliferation (57). EGCG can trigger cell growth arrest pathways at the G1 stage of cell cycle by regulating p27/KIP1, p21/WAF1/CIP1, cdk4, cdk6, and cyclin D1 (58, 59). Catechin pyrogallol inhibits G2-M transition of human lung cancer cells in cell cycling, resulting in tumor growth suppression (60). In BC T47D cell line, catechin activates the phosphorylation of p38 and JNK/SAPK, and the former suppresses the phosphorylation of cell division control protein 2 (CDC2) and regulates the expression of cyclin dependent kinase (CDK), cyclin A and cyclin B1 proteins, leading to G2 arrest (61). EGCG inhibited the proliferation of human lung cancer cells through targeting the epidermal growth factor receptor (EGFR) signaling pathway (62). EGCG displays anticarcinogenic effects in human BTC cell lines by activating caspase and inducing cell arrest in the sub-G1 phase in cell cycling (63).

Catechins anticarcinogenic effects by inducing apoptosis of various types of tumor cells. EGCG inhibited the growth and induced apoptosis (programmed cell death) of human stomach cancer cell line KATO III (64). EGCG triggers the Fas-mediated apoptosis in LNCaP cells by reducing Fas activity, presumably on the cell surface (65). EGCG induces apoptosis in human prostate cancer cells via changing nuclear morphology and DNA fragmentation (66). The EGCG-induced apoptosis and inhibition of EGCG-mediated Bcl2-associated x protein (Bax) expression were partially blocked by pre-treatment with PD169316 [a p38 mitogen-activated protein kinase (MAPK) inhibitor] (67). Similarly, the EGCG-induced apoptosis, and inhibition of EGCG-mediated expression of p-p38 alpha MAPK and Bax were partially blocked by pre-treatment with SHP-1 inhibitor NSC87877. Therefore, it is considered that EGCG induced apoptosis in NB4 cells *via* the SHP-1-p38 alpha MAPK-Bax cascade (68). EGCG promoted the growth inhibition and apoptosis of nasopharyngeal carcinoma (NPC) cell lines CNE-2 and 5-8F through inhibition of the SIRT1-p53 signaling pathway (69). EGCG induced inhibition of growth migration, invasion and apoptosis of human osteosarcoma OS cells through regulating the Wnt/beta-catenin pathway (70). Copper dynamics and mobilization played an important role in the EGCG-induced cancer cell apoptosis through regulating cellular copper transporters CTR1 and ATP7A (71).

The necrosis is thought to be a result of ATP depletion to a level unsuitable for cell survival (72). EGCG induced necrosis-like cell apoptosis through a caspase-independent mechanism in C2F8, K562, and CML cell lines. Furthermore, EGCG promoted cell death induced by imatinib (*p* < 0.01), leading to increased K562 cell death and suppressing the imatinib-resistant viability of cell line K562 (*p* < 0.01) (72). Green tea extract induced necrosis of cancer cell lines K-562U-937, Clone E6-1, and Jurkat, with half maximal inhibitory concentrations, with IC50 = 88 ± 1.89, 98 ± 1.96, and 205 ± 2.23 μg/mL, respectively (73). Topical (+)-catechin prevents DMBA/TPA-induced carcinoma of the skin squamous cells by modulating antioxidants and tumor necrosis factor α (TNF-α) in BALB/c mice (74). ECG reduced tumor viability and induced necrosis in HT29 cells by inactivating glucose-6-phosphate dehydrogenase and transketolase, the key pentose phosphate pathway enzymes (75).

Combination treatment of EGCG with a low energy ultrasound and a low strength pulsed electric field (PEF) for 72 h induced autophagy of human pancreatic cancer cell PANC-1 (76). Autophagy and apoptosis of glioblastoma cells were strongly induced by 500 μM EGCG treatment (77). Also, EGCG induced autophagy by activating light chain 3 (LC3) and generating reactive oxygen species (ROS) in tumor cells. By enhancing autophagy, the sensitivity of cancer cells had been improved, that’s how EGCG can improve the efficacy of anti-cancer drugs (78).

(−)-Epigallocatechin gallate is crucial factor in governing secretion of α-fetal protein (AFP) modulating autophagic activity of cells HepG(2). EGCG reduced AFP secretion and simultaneously induce AFP aggregation in human HCC HepG(2) cells. EGCG-stimulated autophagy induces the degradation of AFP aggregates in HepG(2) cells, and it directly interacted with protein LC3-I, leading to exposure of the eminent Gly-120 site of LC3-I to the other pivotal binding partners such as promoting the synthesis of LC3-II and 1,2-distearoyl-sn-glycero-3-phosphoethanolamine (79). EGCG induced cisplatin-resistant oral cancer CAR cell autophagy in a time- and concentration-dependent manner, during which the AKT/signal transducer and activator of transcription 3 (STAT3) pathway was stimulated and the expression of multidrug resistance 1 (MDR1) was dose-dependently inhibited, suggesting the alteration of AKT/STAT3 signaling and downregulation of MDR1 were partially responsible for the EGCG-induced CAR cell autophagy and apoptosis (80). EGCG promoted nuclear factor-erythroid 2-related factor 2 nuclear translocation and autophagy, enhanced the sensitivity of CRC cells to radiation and inhibited CRC cell proliferation (81).

### Antioxidant and free radical scavenging

Reactive oxygen species is a crucial signaling substance that play important roles in carcinogenesis. ROS is a group of highly sensitive molecules containing oxygen which are continually generated by metabolizing organelles such as endoplasmic reticulum, peroxisomes and mitochondria (82). ROS appears to elicit both pro-malignant and anti-malignant effects with an abnormal spectrum of actions. Excessive accumulation of ROS in cells activates the signaling pathways precipitating DNA damage or inducing mutagenesis, resulting in carcinogenicity. Regulation of ROS levels in cells seems to be an encouraging therapeutic measure, especially for screening anticarcinogenic effects of natural products with strong antioxidant properties (83).

Antioxidants are substances that protect interior molecules from oxidative damages (84). Catechins play a role as reducing agent antioxidants in many reactions owing to their abundant hydroxyl groups (85), and have been attracting enormous attention as possible compounds to be used in prevention and treatment of ROS-targeted cancers. The mechanisms underlying the anticarcinogenic potential of catechins include chelating trace metals, inhibiting oxidases responsible for producing the superoxide anion, scavenging ROS, and activating antioxidant enzymes (86, 87). During the DPPH scavenging behavior, an electron on B-ring in catechins molecules is oxidized by DPPH radical, which generates a catechin phenoxyl radical to be tautomerized to a corresponding o-quinone, then comes the nucleophilic attack by the reactive C-8 or C-6 carbon of other catechins molecules in a Michael-type addition reaction to a quinone on B-ring. Both B-ring and A-ring, but not the gallate moiety, can be the antioxidant site of catechins (87–89). The oligomers of C, EC, and resveratrol have shown anticarcinogenic properties on T24 human urinary bladder cancer cells due to scavenge ROS and chelate metal ions Fe^2+^ and Cu^2+^, the antioxidant capacity of which is significantly higher than their preceding monomers (90). *In vitro* study showed catechin increased manganese superoxide dismutase (MnSOD) gene expression after being incubated with pheochromocytoma cells (PC-12) for 2 days, which can lead to growth inhibition of the cancer cells (91). EGCG can also covalently interact with cysteinyl thiol residues in protein molecules *via* autoxidation, resulting in functional modulation which may be related to the anticancer effects (92).

There was evidence showing EGCG attenuates DNA damage mediated by ROS in pre-malignant cells, resulting in inhibition of tumor initiation (93). These effects were confirmed in cells hepatocytes, lymphocytes, and colonocytes (94, 95). Carcinogen-induced activation of NOX1/ROS is a signaling pathway in malignant and pre-malignant cells, and treating these cells using EGCG markedly suppressed NOX1/ROS signaling and interrupted the carcinogenesis (96). ROS inhibited MAPK phosphatases, which negatively regulate MAPK, it can be speculated that decreasing ROS levels and subsequent phosphorylation blocking of extracellular signal-regulated ERK1/2 (kinases 1 and 2) contribute partially to the antioxidation activity of EGCG in treating and preventing fibrosarcoma (97).

### Inhibition of metastasis of cancer cells

**FIGURE 2:**
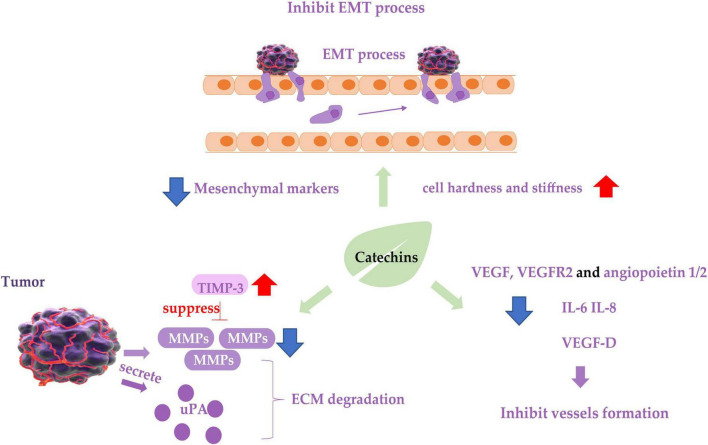
Diagram of catechins inhibiting cancer cell metastasis. Blue arrows indicate downregulation and red arrows indicate upregulation. EMT, epithelial-mesenchymal transition process; TIMP-3, tissue inhibitor of metalloproteinase-3; MMP, matrix metalloproteinases; ECM, extracellular matrix; uPA, urokinase plasminogen activator; VEGF, vascular endothelial growth factor.

The invasion and metastasis of cancer cells are the main reasons for cancer recurrence and treatment failure. The metastatic process of cancer cells includes loss of cell adhesion, increased cell motility and invasiveness, entry into the blood circulation, and spread to distant tissues (98). Several *in vitro* cell experiments and *in vivo* animal experiments have shown that catechins, especially EGCG, could negatively regulate these steps to effectively suppressed the metastasis and invasion of various cancer cells (Figure 2). Specifically, they included inhibiting metastasis by regulating proteolytic enzyme activity, inhibiting the epithelial-mesenchymal transition (EMT) process, and inhibiting the formation of blood vessels or lymphatic vessels.

The anti-metastatic activity of catechins has been confirmed by *in vivo* experiments in mouse models. Different doses of EGCG given by gavage administration inhibited liver and lung metastasis of colon tumors implanted orthotopically in the cecum of nude mice to different degrees, high-dose EGCG had a potent inhibitory effect (99). A study using an *in vivo* mouse model of lung metastasis showed that EGCG treatment significantly reduced the size and number of metastatic nodules in the lungs (100).

Matrix metalloproteinases (MMPs) degrade various protein components in the extracellular matrix (ECM) and inhibit the histological barrier of tumor cell invasion, which plays a key role in tumor invasion and metastasis. Studies on renal carcinoma cells 786-0 and ACHN (101), bladder cancer cell SW780 (102), oral cancer cell OC2 (103), three types of NPC cancer cells (104), hepatocellular carcinoma cell LM6 (105), fibrosarcoma cell HT1080 (106), lung cancer cell CL1-5 (107) showed that EGCG treatment downregulated mRNA or protein expression of MMP-2 or MMP-9, and decreased the ability of the cancer cells to metastasize. Tissue inhibitor of metalloproteinase-3 (TIMP-3) downregulates MMPs activity. Abnormal epigenetic silencing of the TIMP-3 has been associated with the carcinogenesis and metastasis of BC. GTPs or EGCG mediated epigenetic induction of TIMP-3 activity and played a crucial role in blocking invasiveness and gelatinolytic activity of MMP-9 and MMP-2 in BC cells (108). The same effect was found in polycystic ovary syndrome (PCOS) mice treated with oolong tea (109).

Urokinase plasminogen activator (uPA) and its inhibitor plasminogen activator inhibitor-1 (PAI-1) are also associated with ECM degradation and are considered biomarkers for metastasis of cancer cells. GTPs inhibited the uPA secretion and suppressed the metastatic behavior of BC cell malondialdehyde (MDA)-MB-231 by inhibiting activator protein-1 (AP-1) and nuclear factor κB (NF-κB) (110), in which an EGCG derivative 3e was able to inhibit the expression of both uPA and PAI-1, resulting in inhibition of the cancer cell metastasis (111). The inhibitive effect of EGCG on the metastasis of prostate cancer cell LNCaP (112) and the above-mentioned oral cancer cell OC2 (103) is also involved in the inhibition of uPA expression.

Epithelial-mesenchymal transition of cancer cells is considered a prerequisite for the acquisition of an invasive/migratory phenotype and subsequent metastasis. EGCG inhibited the *in vitro* proliferation, migration, and invasion of pancreatic cancer cells, and suppressed mouse xenograft pancreatic cancer *in vivo*. EGCG halted the “Cadherin switch” and suppressed the expression of mesenchymal markers vimentin and β-catenin (113). Similarly, EGCG suppressed the expression of vimentin in non-small cell lung cancer H1299 cells (114), decreased TGF-β1-induced EMT in anaplastic thyroid carcinoma 8505C cells (115), downregulated the expression of EMT phenotypes of cancer stem cells (CSCs) (116), and inhibited these cancer cells invasion and migration. Catechins containing galloyl moiety, or gallated catechins (such as EGCG, GCG, ECG, and CG) potently suppressed the EMT and migration of ovarian cancer cell ES-2 induced by TGF-β (117, 118), indicating they play a role in inhibiting the metastasis of cancer cells by regulating EMT markers and inhibiting EMT. In addition to the molecular phenotypes of EMT mentioned above, cell motility and cell stiffness are considered to be the mechanical phenotypes of EMT and are also closely related to the metastatic activity of cancer cells. Lower stiffness of cancer cells is connected with stronger metastatic potential in many types of cancers. EGCG and green tea extract increased the mean level of Young’s modulus of cancer cells, resulting in increased cell hardness and stiffness and enhanced inhibition of cell motility (118, 119), leading to the suppression of cancer cell metastasis.

Cancer cell metastasis requires angiogenesis and lymphangiogenesis, which involve hematogenous and lymphatic metastasis of cancer cells, respectively. The inhibitory effect of EGCG in synergy with TNF-related apoptosis-inducing ligand (TRAIL) on migration and invasion of prostate cancer LNCaP cells was achieved by inhibiting the protein expression of vascular endothelial growth factor (VEGF) and angiopoietin 1 and 2 (112). VEGF-D is one of the major lymphangiogenic secretory factors, and it induced lymphatic invasion and metastatic spread of cancer cells (119). EGCG treatment reduced VEGF-D secretion in inflammatory breast cancer cells SUM-149 and SUM-190, resulting in reduction of lymphatic endothelial cell migration and tube formation, leading to inhibition of metastasis of cancer cells (120). EGCG has also been shown to reduce the expression of vascular endothelial growth factor receptor 2 (VEGFR2) in CRC cell line RKO (121). Interleukin IL-6 and IL-8 are confirmed to promote angiogenesis in tumor tissues, and treatment of tumor-bearing mice using EGCG suppressed the expression of IL-6 and IL-8 in a concentration-dependent manner, suggesting that IL-6 and IL-8 might mediate the anti-metastatic activity of EGCG (122). EGCG inhibited the angiogenesis of human thyroid cancer xenograft tumors in nude mice (123).

### Regulation of immunity

**FIGURE 3:**
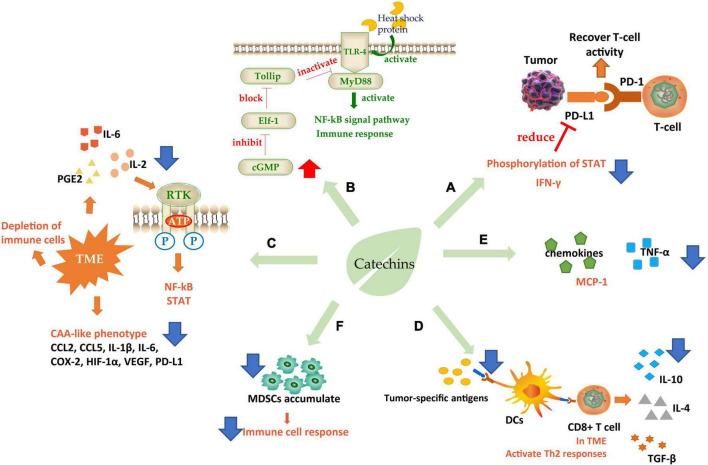
Schematic diagram of catechins regulating immunity. **(A)** Increase immune checkpoint function; **(B)** interaction with TLR-4; **(C)** ameliorate TME; **(D,E)** regulates immune cell response; **(F)** regulated immune cytokines. TLR-4, toll-like receptor 4; TME, tumor microenvironment; DCs, dendritic cells; CAA, cancer-associated adipocyte; MDSCs, myeloid-derived suppressor cells. Blue arrows indicate downregulation and red arrows indicate upregulation.

The immune response to abnormal cells in the body is crucial for maintaining homeostasis. The development of cancer usually triggers a series of immune responses. Tumors can escape immune system attack and migrate by regulating the production of cytokines. Therefore, bioactive substances that target the immune system often have unexpected effects on cancer control, among which catechins are included. Meanwhile, catechins have a significant regulatory effect on multiple sites in the immune system, which has an innate advantage in tumor immunotherapy (Figure 3).

The attack on immune checkpoint is one of the main strategies of tumor cells to escape. The immune checkpoint consists of programmed death factor 1 (PD-1), a receptor molecule secreted by T-cells, and programmed death factor ligand (PD-L1) secreted by tumor cells on the cell membrane. The binding of PD-1/PD-L1 can inhibit the activity of cytotoxic T-cells (CTLs) and even cause T-cell failure, thus protecting tumor cells from immune system attack (124). PD-L1 was induced by epidermal growth factor (EGF) and interferon γ (IFN-γ). Studies were showing that EGCG could regulate the expression of PD-L1 by inhibiting the phosphorylation of STAT and preventing the Janus kinase (JAK)-STAT signaling pathway from activating the transcription factor of PD-L1/PD-L2 (125). PD-L1 mRNA level can also be controlled through the EGFR-Akt signaling pathway (124). IFN-γ is an important factor to stimulate cell expression of PD-L1, so the inhibition of EGCG on IFN-γ could effectively reduce PD-L1 (126). These effects eventually relieve the inhibition of T-cells and improve their attack effect on tumors (Figure 3A).

In addition to PD-1, toll-like receptor 4 (TLR-4) is also a catechin-acting receptor molecule, which may be a target for inhibiting chronic inflammation. Studies have shown that 20% of cancer-related deaths were directly due to TLR-induced cancer cachexia, in which cancer cells released heat shock proteins that acted as TLR-4 agonists in macrophages, skeletal muscle, and fat cells, causing downstream signal transduction. EGCG could effectively downregulate the TLR-4 signal pathway (127). Toll interaction protein (Tollip) is a strong suppressor of this signaling pathway and can directly interact with the ligand IL-1R or TLR4 to inhibit the TLR-induced immune response and negatively regulate signal transduction. Elf-1 is the blocking protein that can inhibit Tollip expression. EGCG achieved the inhibitory regulation of TLR by increasing the level of cGMP in PP2A/cGMP pathway in macrophages to inhibit Elf-1 expression (128). It has also been found that EGCG could inhibit TLR-4/MyD88-mediated NF-κB activation by downregulating both mRNA and protein levels of MyD88 (129) (Figure 3B).

There is an abnormal tumor microenvironment (TME) in cancer patients that causes different immune responses. This low-grade inflammatory state, activated under long-term, low-dose stimulation of specific antigens, often leads to the depletion of immune cells and induces immune tolerance of cancer cells (130). Therefore, some inflammatory factors such as interleukin-2 (IL-2), interleukin-6 (IL-6), and PGE2 are needed to initiate immunosuppression, antagonize this low-grade inflammation and improve the attack of lymphocytes on tumors. EGCG could effectively inhibit inflammation by interrupting the binding of pro-inflammatory factors to receptors and blocking signal transduction such as NF-κB and STAT (131). And the cancer-associated adipocyte (CAA)-like phenotype generated by cells in human triple-negative breast cancer (TNBC) in response to TME. CAA induced cells to secrete many pro-inflammatory factors including CCL2, CCL5, IL-1β, IL-6, and some immune modulators such as COX-2, HIF-1α, VEGF, and PD-L1, which caused carcinogenic through pro-inflammatory effects. EGCG could effectively ameliorate CAA by inhibiting the expression of CAA-related genes and blocking the activation of Smad2 and NF-κB, to alleviate these pro-inflammatory factors and further induces cancer progression (132) (Figure 3C).

Another target of catechins is to regulate immune cell response. First, dendritic cells (DCs), the largest antigen-presenting cells in the immune system, recognize tumor-specific antigens and present them to T-cells (Figure 3D). T-cells initiate a series of responses, typically a Th1 response. Th1-polarized CD4+ T-cells secreted IL-2, IFN-γ, and TNF-α. Activated by IFN-γ and TLRs, macrophages transformed into M1-like macrophages, which can secrete ROS, TNF-α, IL-12, and Nitric oxide (NO) to kill the abnormal cells. At the same time, the cytokines secreted by CD4+ T could further enhance the cytotoxic activity of macrophages and the antigen presentation function of DCs. However, in the TME, Th1 responses were converted to Th2 responses. In this case, Th2 cells secreted IL-4 and IL-10, meanwhile, TGF-β was expressed in large quantities. Simultaneously Foxp3+ B regulatory (Breg) cells and T regulatory (Treg) cells were recruited to initiate humoral immunity. At the same time, m2-polarized macrophages participated in the response to secrete VEGF, IL-10, and TGF-β, which promotes angiogenesis and migration of cancer cells (133, 134). A clinical grade catechin mixture, Polyphenon E, containing about 50% EGCG interacts with the 67 kD laminin receptor (67LR) and promotes the release of granulocyte colony-stimulating factor (G-CSF). The G-CSF then induces the molecular and phenotypic maturation of myeloid-derived suppressor cells (MDSCs) and suppresses their migratory capacity, leading to markedly suppression of their immunosuppressive functions. It was considered that the Polyphenon E could potentially be beneficial to cancer patients through antagonizing cells which interfere with immunotherapy induced antitumor immune responses (135).

(−)-Epigallocatechin gallate plays an efficient regulatory role in this process (Figures 3D,F). EGCG to downregulate the expression of genes necessary for DC presenting antigen, induce the secretion of immunosuppressive factor IL-10, prevent cell surface molecules from binding to receptors, inhibit the low-grade inflammation and prevent the consumption of immune cells (134). Furthermore, EGCG could upregulate the G-CSF secreted by mononuclear MDSCs, a cytokine stimulating the directed proliferation and differentiation of hematopoietic stem cells (133). It also significantly inhibited the accumulation of MDSCs, leading to restoration of the IFN-γ level inhibited by MDSCs, enhancement the activity of CD8+ T-cells, and improvement of the ratio of CD4(+) to CD8(+) T-cells (136), which is beneficial to the improvement of the immune system’s attack on tumor cells. In addition, a phytochemical mixture including EGCG could exert anti-tumor activity by repolarization of m2-polarized macrophages and induce the production of IL12, which could recruit CTLs and natural killer cells (NK) inside tumor cells (137). The combination of EGCG and vitamin A/E increased the adaptive B-cell activity by enhancing adaptive antibody responses and improving CD4(+) and CD8(+) T-cell responses, in which TNF, Il-6, and IL-17 were decreased but IL-15 increased (138). There were studies revealing that EGCG could induce lymphocyte migration in the presence of macrophages, suggesting that the anticancer effect of EGCG depended on the existence of the immune system to a certain extent (139).

It is shown that catechins could act on most of the pathways related to immune regulation (140), among which the star molecules NF-κB and STATS were the targeting signaling pathway of EGCG. EGCG contributes to immunosuppression by inhibiting NF-κB and STATS phosphorylation activation and blocking nuclear translocation to inhibit downstream gene expression (129, 136, 141). EGCG targeting Arg-1/iNOS/NOX2/NF-κB/STAT3 signaling pathway has also been demonstrated in detail, and its activation can enhance immune function (136). Molecular docking experiments proved that catechin has high affinity hydrogen binding ability with NF-κB p52 and TNF-α active site, but has no H-binding effect with anti-inflammatory factor IL 10 (142). Interestingly, EGCG may inhibit the ERK/JUK pathway rather than the p38 MAPK pathway by inhibiting the AP-1 in stimulated human T-cells. EGCG selectively activates signaling pathways (143).

In addition, EGCG also regulated many immune cytokines. For example, TNF-α, an important pro-inflammatory factor in the body, which activates endogenous inflammatory cascades. EGCG antagonized inflammation induced by TNF-α by suppressing the expression of pro-inflammatory cytokines IL-12, IL-6, IL-1β, IL-1α, inflammatory enzymes COX-2 and iNOS, and stimulating anti-inflammatory cytokines IL-10 and IL-4 expression (144). It has also been proved that tea polyphenols could reduce the destruction of the keratinocyte tight junction barrier induced by TNF-α (145). Tea catechins could significantly reduce the expression of TNF-α (142, 143, 146). Chemokines are also a kind of cytokines related to immune cell metastasis. After receiving the antigen information, the immune cells with a specific response will make directional movement along the concentration gradient of the chemical stimulus, which is the chemotaxis of immune cells, and the chemical stimulus that plays locating role is called chemokine. EGCG reduced the migration of neutrophils induced by the chemokine IL-8, inhibited neutrophils recruitment at inflammatory sites, and reduced the expression of pro-inflammatory factors such as MCP-1 (chemokine monocyte chemotactic protein 1) and its receptor (CCR2) (147). Other catechin components have been shown to have immunosuppressive activity. EC could inhibit cell proliferation by inhibiting the AMPK pathway, reducing Akt phosphorylation and mechanistic target of rapamycin (mTOR) expression (148). Like EGCG, EC reduced NF-κB p65 phosphorylation and blocked nuclear translocation, as well as inhibitor levels. By lowering the phosphorylation levels of MAPK members such as P38, JNK, and ERK1/2, EC blocked the signaling pathway (149). EC could inhibit the upregulation of TLR4 and activation of NOX to inhibit downstream events and achieve a preventive effect (150). Immunoactivity of ECG has also been reported, suggesting that ECG inhibited lymphocyte proliferation in an apoptosis-specific manner (151).

In general, catechins maintain the balance of the immune system and have a significant antagonistic effect against most abnormal responses caused by tumors. Catechins have bilateral regulatory effects, i.e., inhibiting the overreaction of the immune system and restoring the immune tolerance caused by tumor cells, in which secretion of cytokines is regulated by their targeting key signaling pathways to maintain a stable and orderly operation of the intracellular immune system.

### Interaction with anticancer drugs

The advent of a variety of anticancer drugs can be beneficial to controlling cancers to a certain extent, resulting in an improvement in life quality for the patients. However, problems in clinical chemotherapy still exist, such as low bioavailability, cell resistance, and strong toxic and side effects, which greatly limit the use of the drugs. Studies were showing that catechins would play a role in alleviating these side effects induced by chemotherapy, owing to their diversely biological activities including antagonistic effects on oxidative stress. Due to their effects on the important signaling pathways *in vivo*, catechins are often used as sensitizing agents in combination with drugs. The multiple phenolic hydroxyl groups in catechins can bind with various proteins through hydrogen and ionic bonds, regulating the functions of the proteins. The combination of anti-cancer drugs with catechins, whether before or after drug administration, would alleviate the toxic and side effects induced by the drugs to a certain extent, resulting in apoptosis acceleration of cancer cells and efficacy enhancement of the drugs.

#### Interaction with cisplatin

Cisplatin is a platiniferous anticancer drug, which induces apoptosis of cancer cells by inhibiting their mitosis via binding directly to DNA and interfering with DNA replication and repair processes. But this is accompanied by extensive drug toxicity.

The combination of cisplatin with EGCG will improve the chemotherapeutic sensitivity of cells to the drug, leading to a reduction of cisplatin dosage. Copper transporter 1 (CTR1) is a transporter related to the intake of cisplatin. EGCG improved the sensitivity of cancer cells to cisplatin by stimulating the expression of CTR1. Specifically, EGCG inhibited ERK1/2, accompanied by enhancement of expression Nuclear enriched abundant transcript 1 (NEAT1) and CTR1 through ROS initiation (152). NEAT1 is a key factor inducing CSCs, and it causes cellular resistance to cisplatin. CTR1 and NETA1 are mutually regulated. EGCG-induced upregulation of expression in NEAT1 would increase CTR1 expression (153), which in turn inhibited CSCs regulated by NEAT1, leading to the reverse stemness induced by NEAT1 (154). Also, EGCG inhibited the CSCs phenotypes and reduced the resistance of cancer cells to drugs via increasing the expression of miRNA 485, an upstream gene for CD44 in cisplatin-resistant cells (155).

(−)-Epigallocatechin gallate is an inhibitor of some enzymes, including 5′-3′ structure-specific endonuclease excision repair cross-complementation group 1/xeroderma pigmentosum group F (ERCC1/XPF). ERCC1/XPF is an enzyme involved in repairing DNA damage induced by cisplatin. EGCG enhanced the sensitivity of cancer cells to cisplatin via targeting ERCC1/XPF, leading to the inhibition of DNA repair of the cancer cells (156).

(−)-Epigallocatechin gallate promoted cisplatin-induced cancer cell apoptosis and enhanced the therapeutic effect of cisplatin. Specifically, EGCG played the synergistic action through upregulation of the p19Arf-p53-p21Cip1 signaling pathway that plays a crucial role in regulating cell proliferation. Murine double minute 2 (MDM2) is a tumor suppressor which promotes ubiquitination and degradation of p53 whose expression is activated via inhibition of MDM2.

P19 inhibits MDM2 activity by binding to it. Compared with cisplatin alone, the combination of cisplatin with EGCG increased the expression of P19, P53, and P21 at both mRNA and protein levels, leading to cell cycle arrest in G1 phase and promoting apoptosis of cancer cells (157). Furthermore, EGCG-induced inactivation of NF-κB could prevent migration and self-renewal of cancer cells by coordinating the cell localization of NF-κB p65 and the transcriptional level of TWIST 1 (158).

(−)-Epigallocatechin gallate, with its excellent anti-inflammatory and antioxidant activity, can effectively ameliorate the toxic and side effects of cisplatin. The oral administration of EGCG could not only resist cisplatin-induced inflammation, reduce the production of apoptotic proteins and inhibit cell damage, but also improve the toxic effects of cisplatin-induced oxidative and nitrosative stress by enhancing the total antioxidant capacity (159, 160). EGCG downregulated NF-κB and upregulated nuclear factor-erythroid related factor 2 (Nrf2) and heme oxygenase 1 (HO-1), resulting in enhancement of anti-inflammatory effect. Meanwhile, EGCG itself could increase the activity of Caspase3/9, downregulate MDR1, regulate the AKT/STAT signaling pathway, and induce apoptosis and autophagy, which increases the cisplatin drug potency (80). When cisplatin was used with EGCG, acidic vesicular organelles and intracellular microtubule-associated protein 1 light chain 3 (LC3-II) in the cytoplasm were accumulated, accompanying an increase in autophagosomes, indicating that EGCG synergically inhibited cell proliferation and induced cell apoptosis by increasing autophagy (161).

#### Interaction with doxorubicin

Doxorubicin (DOX) is a widely used antibiotic drug that can kill multiple tumors, but it has serious toxic effects on healthy cells, such as DOX-induced ROS oxidative stress. EGCG scavenged ROS by improving the activity of antioxidant enzymes such as MnSOD, suppressing the depletion of the antioxidant glutathione and inhibiting MDA level, resulting in increased vitality of the healthy cells exposed to DOX (162). Administration of EGCG after DOX treatment could enhance the expression of EGFR family ErbB2 to promote cell growth, but reduce the activity of caspase 12 and calpain 2, downregulating the expression of NF-κB p65 subunit and the downstream genes in the apoptosis pathway, during which the anticancer efficacy of DOX was increased and its IC50 on cancer cells decreased (162). EGCG administration prior to DOX could effectively reduce pro-inflammatory factors TNF-α, NF-Kappa B, and iNOS induced by DOX (163).

In addition, EGCG can also reduce cancer cell resistance to DOX. Studies have shown that EGCG significantly suppressed the p-AKT and ERK by downregulating the expression of downstream STAT, making the cells more sensitive to the growth inhibition and apoptosis induced by DOX (164). Meanwhile, EGCG could inhibit DOX-induced overexpression of P-gp (165), an ABC transporter acting as an energy-dependent “drug pump” that excludes the drugs from the cells, reducing intracellular drug concentrations and resulting in drug resistance. EGCG could maintain the drug level in cancer cells by binding to P-gp (166) and promote apoptosis by reducing DOX-induced pro-survival autophagy (167). Further studies showed that EGCG interacted with DOX by reducing LncRNA SOX2OT variant 7, inactivating the Notch3/DLL3 signaling pathway targeting this RNA, leading to a reduction in the stemness of cancer cells and inhibition of their drug resistance (168).

#### Interaction with erlotinib

Erlotinib is an EGFR tyrosine kinase inhibitor that inhibits EGFR phosphorylation. Studies have shown that EGFR kinase domain mutations are happened in cancer cells, which induces ligand-independent phosphorylation and activates the downstream uncontrolled signal transmission. Both Erlotinib and EGCG could bind to the ATP binding pocket of EGFR, resulting in inhibition of the phosphorylation (169) and a synergistic effect with Erlotinib. Erlotinib stabilizes EGF on the plasmalemma, however, EGCG induced internalization and ubiquitination of EGF, resulting in disruption of EGFR signal transduction (170).

(−)-Epigallocatechin gallate synergistically inhibited Erlotinib-induced inhibition of cell cycle suppressors p21 and p27, leading to enhancement of the cell cycle arrest, during which the expression of apoptosis regulatory protein Bim was not affected. Protein p53 is a factor influencing the EGCG and Erlotinib induced growth inhibition by mediating the NF-κB signaling pathway and its downstream transcription target B-cell lymphoma-2 (Bcl-2). A combination of EGCG and Erlotinib could improve the expression of p53 (171). Further study showed that EGCG and its combination with Erlotinib had no significant effects on the mRNA expression of Bcl-2, p27, p21, or Bim, suggesting they are post-transcriptional regulation (172). The two drugs’ combination facilitated normal cell proliferation and suppressed tumor growth rate (173). Clinical trials on phase IB patients showed that the combination of GTPs and Erlotinib showed a high rate of pathological reaction and good cancer-free survival (CFS), suggesting that the adjuvant therapy of EGCG has a certain clinical promotion value (174).

#### Interaction with docetaxel

Docetaxel (DOC), a derivative of Taxanes, is a class of anticancer drugs targeting hormonal tumors. The hormonal tumors, such as prostate and BC, are largely caused by the uncontrolled binding of hormones to corresponding receptors. DOC produces aromatase inhibitors, leading to changes in transcriptional activity and nuclear localization of androgen receptors, and tumor inhibition. EGCG strengthened the inhibitive effect of DOC on the PISK/Akt signaling pathway, the transduction and activation of STAT3, and the expression of MDR protein, leading to inhibition of cancer cell invasion and metastasis (175). EGCG and DOC bound to different locations of tubulin, showing synergistically suppressive effects on the targeted tubulin, causing cell cycle arrest (176). Low-dose metronomic (LDM) chemotherapy showed that EGCG targeted the angiogenesis of normal cells and reduced the side effects of DOC (177). Furthermore, a combination of DOC in the EGCG significantly improved the absorption and transport of the anticancer drug DOC (178).

#### Interaction with irinotecan

Irinotecan (IRN) is a topoisomerase I inhibitor that inhibits tumor progression by targeting topoisomerase to damage DNA structure. The combination of IRN with EGCG could simultaneously improve the bioavailability of IRN and reduce its side effects. EGCG collaborated with IRN to cause more extensive DNA damage to arrest cancer cell cycle in the S/G2 phase and promote autophagy (179). Furthermore, EGCG enhanced the bioavailability of IRN by inhibiting the ATPase activity of drug pump P-gp, resulting in significant suppression of the bile efflux of IRN and SN-38, the active metabolite of IRN that prevented the drug efflux into the biliary elimination, leading to significantly prolong the drug’s half-life in plasma (180). Due to the antioxidant properties of catechins, the combination could effectively reduce IRN-induced toxicity, including ameliorating symptoms such as diarrhea and leukopenia, along with reduction of adenoma and non-alcoholic fatty liver disease (NAFLD) (181). EGCG could also reduce IRN-induced inflammatory factors and inhibit ROS production, and restore MMP-2 and MMP-9 reduction induced by IRN, protecting the oral mucosa against deleterious effects (182).

#### Interaction with daunorubicin

Daunorubicin (DNR) is a powerful anticancer drug, but it has serious cardiotoxicity. Carbonyl reductase 1 (CBR1) metabolizes DNR into Daunorubicinol, which reduces the anticancer activity of DNR and increases its cardiotoxicity. Catechins, especially EGCG, inhibited the catalytic activity of CBR1 by directly binding to its active site, reducing the side effects of DNR (183). The transport of DNR is P-gp dependent, and catechins would interact with the allosteric site of P-gp to improve its holistic function and transport efficiency, and ultimately inhibited DNR outflow, improving the intracellular DNR level (184). EGCG could bind to 67LR and activate PP2A through EGCG/67LR/PKA/PP2A pathway, leading to the dephosphorylation of subunit MYPT1 of myosin phosphatase, making THP-1 leukemic cells more sensitive to DNR and promoting apoptosis (185).

#### Interaction with gemcitabine

Gemcitabine is a cytosine nucleoside derivative, which is activated by deoxycytosine kinase and metabolized by cytosine nucleoside deaminase after entering the body. It is a drug that directly acts on genes through the incorporation of major metabolites into DNA and influences the G1/S phase. EGCG played a synergistic role with Gemcitabine through the inhibition of signaling pathways. EGCG decreased the phosphorylation levels of signal transduction factors such as AKT, ERK, JNK, etc., which weakened cell stress response to stimulation caused by treatment and improved cell sensitivity, leading to an increase in drug effects (186). STAT3 seemed to be a key acting site that enhanced the inhibition of cell viability by EGCG, while Gemcitabine could inhibit the target gene of STAT, showing a synergistic effect with EGCG (164, 187).

Epithelial-mesenchymal transition is a marker of cancer metastasis. The combination of Gemcitabine with EGCG inhibited the growth, invasion, and migration of pancreatic cancer cells partially *via* the suppression of EMT and Akt pathway, resulting in enhancement of Gemcitabine efficacy (114). Tumor suppressor factors such as DAPK2 and decoy receptor of RANK ligand OPG were upregulated by EGCG. The increased expression of OPG suppressed the activation of NF-κB induced by the RANK receptor, making cells more prone to apoptosis (188).

#### Interaction with 5-fluorouracil

The 5-fluorouracil (5-FU) is a thymidylate synthase inhibitor that inhibits the methylation of deoxyuridine acid (dUMP) into deoxythymidylate (dTMP), affecting DNA synthesis. Green tea extract or EGCG could improve the half-life of 5-FU (189), enhancing its growth inhibition, and reducing the viability and migration of cancer cells. Moreover, studies have shown that EGCG increased the number of cells in G2/M phase, while 5-FU blocked the cell cycle in the S phase (190). On the other hand, EGCG could enhance 5-FU resistance by inhibiting the expression of P-GP and receptor VEGF (191). Furthermore, EGCG targeted the ATP binding domain of glucose-regulated protein 78 (GRP78), preventing it from exercising its protective function and increasing the sensitivity of cells to drugs (192). Inhibition of GRP78 activates the NF-κB pathway, leading to a series of downstream responses, for example, which increase mir-155-5p targeted MDR1 expression which suppresses efflux of 5-FU, leading to the accumulation of 5-FU in cancer cells, activation of Caspase-3 and PARP, and decrease in Bcl-2 but increase in Bad, which in turn causes the cancer cells apoptosis (193).

#### Interaction with sulindac

Sulindac is a non-steroidal anti-inflammatory drug, which can inhibit inflammatory factors *in vivo*. EGCG could prevent lipid peroxidation, downregulate the expression of iNOS, and effectively reduce intracellular oxidative damage. When combined with Sulindac, EGCG improved the anti-inflammatory activity of Sulindac by increasing the activity of caspase-3, and so induced cell apoptosis, promoting therapeutic effects of Sulindac (194). The combination of Sulindac and green tea catechins upregulated RARα1, but downregulated MAP3KI4 (NF-κB induced kinase), death-related protein kinase I (DAPK1), and tyrosine protein kinase (SKY), inhibiting the NF-κB pathway and blocking important signaling in cancer development, though GADD153, a key transcription factor inducing apoptosis, and P21, a protein blocking cell cycle and inhibiting proliferation, were not significantly upregulated when the two drugs were used individually (195).

Aberrant crypt foci (ACF) is a new precancerous lesion that occurs in the early stage of CRC. The combination of EGCG and Sulindac could synergistically promote cancer cell apoptosis by inhibiting the formation of ACF and alleviating the side effects caused by Sulindac (196).

#### Interaction with gefitinib

Gefitinib is another tyrosine kinase inhibitor, and its combination with EGCG could enhance the inhibition of tumor migration and invasion by decreasing enzymatic activity of tyrosine kinase and the expression of MMP-2, and suppressing the phosphorylation of ERK, JNK, p38, and AKT. Most importantly, EGCG administration inhibited the phosphorylation of EGFR, making the cancer cells more sensitive to Gefitinib (197). An epigallocatechin gallate derivative (EGCGD) isolated from Anhua dark tea presented cancer cells from resistance to Gefitinib through suppressing EMT and PI3K/mTOR signal pathway (198).

#### Interaction with temozolomide

Temozolomide (TMZ) is a DNA-methylating drug. O-6-methylguanine DNA-methyltransferase (MGMT) is the key factor for cell resistance, thus maintaining a low level of MGMT expression is more conducive to the efficacy of TMZ. EGCG could inhibit the expression of MGMT. EGCG alleviated the resistance of MGMT to TMZ and then enhanced TMZ cytotoxicity to cancer cells by inhibiting β-catenin transfer into the nucleus and blocking the expression of downstream genes (199). EGCG degraded PARP by downregulating P-Akt, Bcl-2, and P-gp, suppressing the vitality of drug-resistant cancer cells and decreasing the stemness of tumor cells, resulting in more sensitivity of cancer cells to TMZ and apoptosis (200).

Glucose-regulated protein 78 plays an important role in protein folding and assembly. Cell surface GRP78 is found to act as a receptor or co-receptor for numerous ligands, promoting signaling cascades relating to tumor cell survival and proliferation. EGCG could directly act on the ATP binding domain of GRP78, decreasing the protective function of GRP78, and making cancer cells more sensitive to TMZ (192). The combination of TMZ with EGCG increased the therapeutic efficacy of TMZ in orthotopic mouse glioblastoma models by inhibiting the expression level of GRP78 (201).

Synergistic effects of EGCG were found in many other anticancer drugs. EGCG could significantly enhance the inhibitory effect of Safingol (a competitive inhibitor of SPHK1) on CLL cancer cells. EGCG/SPHK1 inhibitor combinations would be a novel therapeutic strategy for CLL patients with 67LR and SPHK1 overexpression (202). Tegafur, a chemotherapy drug for gastrointestinal tumors, reduces the expression of α-defensin, an important antibacterial peptide in the intestinal innate immune system, and induces the production of ROS. EGCG, when used with Tegafur, inhibited the production of ROS induced by Tegafur and prevented α-defensin expression decline caused by Tegafur, reducing the side effects of drugs (203).

Dacarbazine is an anti-tumor drug that induces cancer cell growth arrest or apoptosis by causing nucleic acid methylation or direct DNA damage. Combination of EGCG and Dacarbazine enhanced the efficacy of Dacarbazine by inhibiting activities of FAK and MMP-9, as well as proliferation and/or metastasis of cancer cells, in which the effective dosage of Dacarbazine was reduced, resulting in less potential cytotoxicity to the healthy cells (204).

Much attention has been paid to the clinical adjuvant therapy of catechins (40). For EGCG, administered after Sunitinib, showed synergistic effects by acting on the IRS/MAPK pathway, in which the effects of Sunitinib on inhibition of proliferation and VEGF secretion were increased. *In vivo* experiments showed that EGCG injection at the 4th hour after Sunitinib administration reduced angiogenesis and inhibited tumor growth, accompanying significant downregulation of IRS-1 levels.

### Regulation of signaling pathways

**FIGURE 4:**
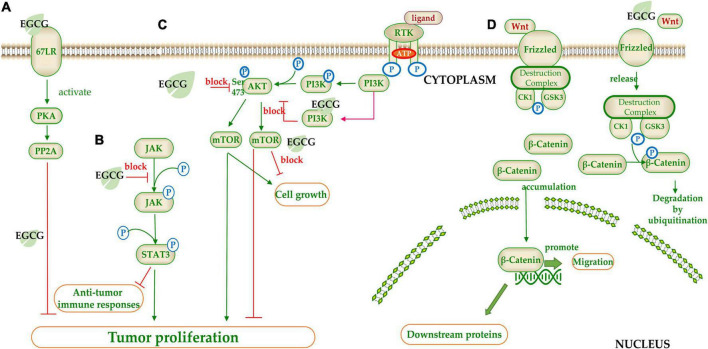
The target of catechins in the signaling pathway. **(A)** 67LR/PKA/PP2A signal pathway; **(B)** JAK/STAT signal pathway; **(C)** mTOR/AKT signal pathway; **(D)** Wnt/β-Catenin signal pathway. The green arrow represents the signal transmission, red arrow represents inhibition.

**FIGURE 5:**
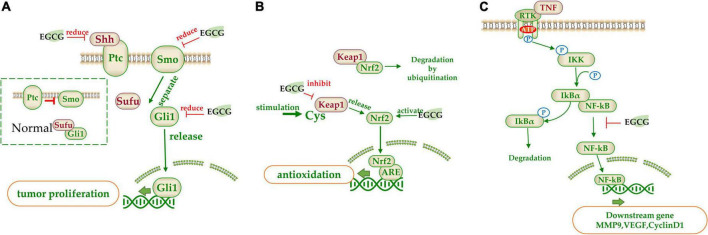
The target of catechins in the signaling pathway. **(A)** Sonic hedgehog (SHH) pathway signal pathway; **(B)** Nrf2 signal pathway; **(C)** NF-κB signal pathway. The green arrow represents the signal transmission, red arrow represents inhibition.

The signaling pathway is the core system in which cells regulate various physiological processes and respond to external stimuli. Normally, cells have a complete set of regulatory mechanisms for initiating and/or inhibiting signal reception, cascade transmission, and ultimately gene expression, but in cancer cells, the signaling pathway is usually overactivated, and the balance is broken. Catechins played an anticarcinogenic role by promoting and/or inhibiting signal transmission through the targeted regulation of multiple links in the signal pathways.

(−)-Epigallocatechin gallate regulates signaling pathways by interacting with membrane receptors. Signal transduction usually begins when an external stimulus activates a receptor on the cell membrane. Membrane target protein 67LR, an important membrane glycoprotein to communicate with the ECM, is widely expressed in tumor cells, which in turn can affect tumor metastasis and regulate many other signal pathways. EGCG matched to 67LR at residues 161–170 (205, 206) and bound to 67LR via lipid rafts (207), blocking the activation of downstream pathways by preventing the combination of the receptors and ligands. In the PKA/PP2A pathway, EGCG binding to 67LR induced the activation of PKA, which dephosphorylates related proteins such as the tumor suppressor Merlin and inhibited the proliferation of cancer cells (208) (Figure 4A). VEGF receptor is a high affinity receptor binding specifically to VEGF. Inactivation of the VEGF signaling pathway suppresses angiogenesis, a common strategy for inhibiting carcinogenesis. EGCG significantly inhibited the expression of VEGF and reduced VEGF receptors (209).

(−)-Epigallocatechin gallate regulates signaling pathways by inhibiting phosphorylation of signaling molecules (Figures 4B–D). STAT is a transcription factor family that combines with DNA to initiate downstream genes transcript. STAT3 suppresses anti-tumor immune responses and promotes the proliferation and migration of cancer cells. EGCG inhibited STAT3 phosphorylation by blocking JAK2 phosphorylation (210) (Figure 4B). The mTOR is a serine/threonine kinase belonging to the PI3K related kinase (PIKK) family, which can regulate cell growth and proliferation (211). EGCG blocked AKT phosphorylation at Ser473, and also acted as an ATP competition inhibitor competing for ATP binding sites of PI3K and mTOR to block the mTOR signal pathway (212) (Figure 4C). MAPK signaling pathway is a core pathway in the cellular regulatory network. EGCG inhibited the MAPK signaling by competing for the phosphorylation sites of downstream proteins (213). Wnt (Wingless-type mice mammary tumor virus integration site family) pathway is a highly conserved signal pathway in species evolution, which plays an important role in early embryo development, organogenesis, and tissue regeneration. The key factor in this pathway is β-catenin, a class of transcription factors that activate the cell division related gene expression. β-catenin binds to the destruction complex when the Wnt ligand does not bind to Frizzled family of proteins (FZD). CK1 and GSK3 in destruction complex can phosphorylate β-catenin, leading to its degradation by E3 ubiquitin ligase. When Wnt is combined with FZD, destruction complex will be confined to the cell membrane and cannot degrade β-catenin, resulting in accumulation of β-catenin in the cytoplasm (214). EGCG could inhibit the Wnt pathway by phosphorylating β-catenin and promoting its degradation (215) (Figure 4D).

(−)-Epigallocatechin gallate suppressed some signaling pathways by reducing the related bioactive proteins (216). Sonic hedgehog (SHH) pathway involves in regulating cell proliferation and differentiation. Excessive activation of SHH leads to carcinogenesis. The downstream transcription factor Gli1 is the key factor of the SHH pathway and it is regulated by membrane receptors Patched (Ptc) and Smoothened (Smo). Normally, Ptc inhibits Smo, and Gli1 binds to the protein kinase inhibitor SUFU and is trapped in the cytoplasm. When SHH binds to Ptc1, the inhibition of Smo is relieved and SUFU is disconnected from Gli1. Then Gli1 will enter the nucleus to initiate the expression of downstream genes (217). EGCG inhibited the expression of Smo, SHH, Gli1, and Gli2 (193) (Figure 5A).

(−)-Epigallocatechin gallate regulates some signaling pathways by interacting with specific inhibitors in the pathway. Nrf2 pathway involves in a variety of diseases and Kelch-like ECH-associated protein 1 (Keap1) is its inhibitor which acts as a bridge between Nrf2 and E3 ubiquitin ligases. When Nrf2 is inactive, it will be degraded by ubiquitination. When Keap1’s key cysteine is modified upon stimulation, it will lose its inhibitive effect and release the Nrf2. The released Nrf2 is then transferred to the nucleus to bind with antioxidant responsive element (ARE), which activates downstream transcription factors. EGCG could inhibit Keap1 and activate Nrf2, resulting in the expression of the downstream antioxidant related genes (218) (Figure 5B). Furthermore, EGCG could induce the re-expression of secreted frizzled-related protein 1 (SFRP1), an inhibitor of the Wnt pathway, which prevents ligand-receptor interaction and is silenced in cancer cells (215).

(−)-Epigallocatechin gallate can regulate some signaling pathways by controlling downstream genes expression in the signal pathway. EGCG could act on the MMP associated with cell apoptosis (207, 219). These enzymes are secreted by tumor cells and can degrade various proteins in the ECM and are the main proteolytic enzymes in tumor invasion and metastasis. EGCG inhibited the activities of MMP2 and MMP9, and promoted the expression of tissue inhibitor of MMPs (TIMp1/2) to suppress the invasion and metastasis of tumor cells (107). Peptidyl-prolyl isomerase NIMA-interacting 1 (PIN1) is a specially phosphorylated prolyl isomerase necessary for mitotic regulation and a direct target of EGCG. EGCG inhibited the activity of PPIase (207) and reduced the expression of PIN, inducing apoptosis (220). NF-κB is normally inactivated when it binds to the inhibitor IkBα. However, when upstream signal factor TNF binds to membrane receptors, IkB kinase (IKK) is activated, which phosphorylates IkBα and dissociates NF-κB. Free NF-κB is released into the nucleus and binds with nuclear DNA to initiate the transcription of genes such as CyclinD1, C-MYC, MMP-9, and VEGF (221). EGCG could significantly downregulate NF-κB activity, inhibiting these gene transcription (222) (Figure 5C).

## Inconsistent results and future expectations

Although there have been a lot of *in vivo* and *in vitro* studies revealing tea catechins had anticarcinogenic effects, inconsistent results were also observed and reported. A 7-year (1995–2001) population-based cohort study in Japan involving 41,440 male and female patients with ages ranging from 40 to 79 years gave no direct evidence showing green tea consumption being correlated to lower risk of lung cancer (223). No significant relationship was found between colon cancer and intake of EC or tea (30, 224). Population based case-control studies showed green tea drinking was no associated a reduced risk of pancreatic cancer (225). A screening study on prostate, lung, colorectal and ovarian (PLCO) cancers involving 57,398 men and women showed that heavy tea drinking was not correlated to risk of overall CRC (RR = 0.77, 95% CI = 0.55∼1.09, *p* = 0.17) or the risks of CRC cancer site (*p* = 0.14) or CRC stage (*p* = 0.60) (226). A cohort study in Japan showed no significant relationship of green tea drinking to the reduced risk of acute myeloid leukemia (227). A 14-year follow-up EPIC (European Prospective Investigation into Cancer and Nutrition) cohort study including 476,160 male and female residents from 10 European countries, among which there were 5,991 incident CRC cases (3,897 colon cancer cases and 2,094 rectum cancer cases), revealed by multivariable-adjusted Cox regression model, that a doubling intake of total dietary polyphenol was not correlated to CRC risk in men (HR = 0.97, 95% CI = 0.90∼1.05) or in women (HR = 1.06, 95% CI = 0.99∼1.14) (228).

What causes the inconsistency in these research findings? The metabolism and/or chemically changing of the in-taken catechins might be important factors influencing the anticarcinogenic effects of tea and catechins. There was a study showing that the in-taken catechins are promptly O-methylated by *COMT* (human catechol-*O*-methyltransferase) and so the association between tea intake and BC depends on *COMT* genotype. Among women who carried minimum one low activity *COMT* allele, the risk of BC in tea drinkers was significantly lower than those who did not drink tea (OR = 0.48, 95% CI = 0.29∼0.77), after balancing the related dietary, menstrual, reproductive, and demographic factors. Among women who carried homozygous high activity *COMT* allele, however, no significant difference in the BC risk was observed between non-tea drinkers and tea drinkers (OR = 1.02, 95% CI = 0.66∼1.60) (18, 20). The risk of lung cancer was decreased by 72% among daily tea drinkers who carried the OGG1 Cys (326) allele (95% CI = 0.09∼0.94). Among people carrying GSTM1 null homozygotes, on significant difference in lung cancer risk was observed between daily tea consumers and tea non-consumers. Green tea drinking showed no effect on the risk of lung cancer among GSTM1, AKR1C3, or OGG1 Ser (326) homozygote carriers. It is considered that the chemopreventive effects of green tea consumption may be limited to the population who are exceptionally susceptible to DNA damages induced by oxidative stress (71, 94, 156).

Interaction of catechins with partial drugs resulted in a reduction in drug bioavailability might lead decrease in the therapeutic efficacy of the drugs. Simultaneous administration of EGCG and anticancer drug Sunitinib would decrease Sunitinib concentration in plasma, thus reducing its therapeutic effect (224) because EGCG has the same binding sites on human serum albumin (HSA) as some drugs, such as DOX and TF (225, 226), leading to less bioavailability of the drugs.

To explore the potentials of tea catechins in anticarcinogenic drug development, the following topics should be focused on: (1) metabolism of in-taken catechins in the gastrointestinal tract and development of methods for protecting the in-taken catechins from degradation or transformation; (2) technology for improving the bioavailability of catechins, such as encapsulation and nanoparticle; (3) novel catechins formulae with synergic anticarcinogenic effects.

## Conclusion

**FIGURE 6:**
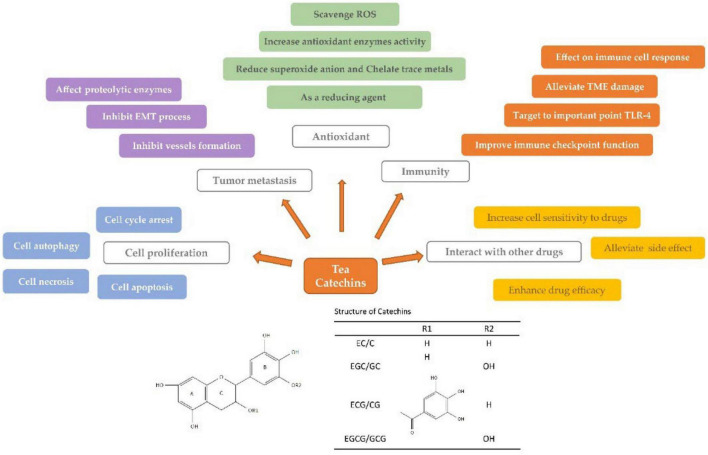
Anticarcinogenic mechanism of tea catechins. Tea catechins exert their anticarcinogenic effects by inhibiting cell proliferation (blue module), preventing tumor metastasis (purple module), reducing oxidative damage (green module), improving immune activity (orange module), and ameliorating the potency of anticancer drugs (yellow module). Different colored textboxes indicate the specific ways in which tea catechins exert.

Epidemiological and *in vivo* studies showed that tea catechins have anticarcinogenic effects on many cancers including gynecological cancers, digestive tract cancers, incident glioma, liver and gallbladder cancers, lung cancer, etc. Catechins suppressed cancers through inhibiting proliferation and metastasis of cancer cells, antioxidation and scavenging free radicals, enhancing body immunity, and synergistically interacting with anticancer drugs, which involved many signaling pathways (Figure 6).

## Author contributions

Y-RL and X-QZ designed the project. Y-RL, X-QZ, and X-XL searched and collected the literature. X-XL wrote the section “Interaction with anticancer drugs” and designed the Figures. CL wrote the sections “Inhibition of cancer cell proliferation and growth” and “Antioxidant and free radical scavenging.” S-LD wrote the section “Inhibition of metastasis of cancer cells.” C-SO wrote the section “Regulation of immunity.” J-LL wrote the section “Regulation of signaling pathways.” J-HY wrote the section “Inconsistent results and future expectation.” Y-RL wrote the sections “Introduction,” “Gynecological cancers,” “Digestive tract cancers,” “Incident glioma,” and “Conclusion.” X-QZ wrote the sections “Liver and gallbladder cancers,” “Lung cancer,” and “Other cancers.”

## Glossary

5-FU: 5-fluorouracil
67LR: 67 kD laminin receptor
ABC: ATP-binding cassette transporter
ACF: aberrant crypt foci
ACHN: human renal cell carcinoma
AFP: α-fetal protein
AKR1C3: aldo-keto reductase family 1 member c3
Akt: protein kinase B
AMPK: adenosine 5′-monophosphate-activated protein kinase
AP-1: activator protein 1
ARE: antioxidant responsive element
Arg-1: arginase 1
ATP: adenosine triphosphate
ATP7A: ATPase copper transporting 7 alpha
Bax: Bcl2-associated x protein
BC: breast cancer
Bcl-2: B-cell lymphoma-2
Bim: Bcl-2 interacting mediator of cell death
BTC: biliary tract cancer
C: (+)-catechin
CAA: cancer-associated adipocyte
CAR: oral cancer cell-line
CBR1: carbonyl reductase 1
CCL: C-C motif ligand
CCR2: C-C motif chemokine receptor 2
CD4: cluster of differentiation 4
CDC2: cell division control protein 2
CDK: cyclin dependent kinase
CFS: cancer-free survival
CG: (+)-catechin-3-gallate
cGMP: cyclic guanosine monophosphate
CI: cumulative incidence
CIP1: cyclin dependent kinase interacting protein 1
CK1: casein kinase 1
CLL: chronic lymphocytic leukemia
COMT: human catechol-*O*-methyltransferase
COX-2: cyclooxygenase 2
CRC: colorectal cancer
CSCs: cancer stem cells
CTL: cytotoxic T-cells
CTR1: copper transport protein 1
DAPK2: death associated protein kinase 2
DCs: dendritic cells
DLL3: delta-like ligand 3
DMBA: 7,12-dimethylbenz(a)anthracene
DNR: daunorubicin
DOC: docetaxel
DOX: doxorubicin
DPPH: 2,2-diphenyl-1-picrylhydrazyl
dTMP: deoxythymidylate
dUMP: deoxyuridine monophosphate
EC: (−)-epicatechin
ECG: (−)-epicatechin gallate
ECM: extracellular matrix
EGC: (−)-epigallocatechin
EGCG: (−)-epigallocatechin gallate
EGCGD: epigallocatechin gallate derivative
EGF: epidermal growth factor
EGFR: epidermal growth factor receptor
Elf-1: ETS transcription factor
EMT: epithelial-mesenchymal transition
EPIC: European Prospective Investigation into Cancer and Nutrition
ER: estrogen receptor
ErbB2: Erb-b2 receptor tyrosine kinase 2
ERCC1/XPF: excision repair cross-complementation group 1/xeroderma pigmentosum group F
ERK: extracellular regulated protein kinases
FAK: focal adhesion kinase
FZD: Frizzled family of proteins
GADD153: growth arrest and DNA damage-inducible 153
GC: (+)-gallocatechin
GCG: (+)-gallocatechin-3-gallate
G-CSF: granulocyte colony-stimulating factor
GLI1: glioma-associated oncogene homolog 1
GRP78: 78-kd glucose-regulated protein
GSK3: glycogen synthase kinase 3
GSTM1: glutathione S-transferase mu 1
GTPs: green tea polyphenols
HCC: hepatic cell carcinoma
HepG(2): human hepatocellular carcinomas
HIF-1α: hypoxia inducible factor-1α
HO-1: heme oxygenase 1
HPV-16: human papillomavirus type 16
HR: hazard ratio
HSA: human serum albumin
IFN-γ: interferon γ
IkBα: inhibitor of NF-κB
IKK: Ikb kinase
IL-1R: interleukin-1 receptor
iNOS: inducible nitric oxide synthase
IRN: irinotecan
IRS: insulin receptor substrate
JNK: C-jun n-terminal kinase
Keap1: kelch like ech-associated protein 1
KIP1: kinesin-like protein 1
LC3: light chain 3
LDM: low-dose metronomic
LNCaP: human prostate cancer cell-line
LncRNA: long non-coding RNA
MAP3KI4: NF-κB induced kinase
MAPK: mitogen-activated protein kinase
MCP1: chemokine monocyte chemotactic protein 1
MDA: malondialdehyde
MDA-MB-231: breast cancer cell-line
MDM2: murine double minute 2
MDR1: multidrug resistance 1
MDSCs: myeloid-derived suppressor cells
MGMT: O-6-methylguanine DNA-methyltransferase
MMPs: matrix metalloproteinases
MnSOD: manganese superoxide dismutase
mTOR: mechanistic target of rapamycin
MyD88: myeloid differentiation primary response protein 88
MYPT1: myosin phosphatase target subunit
NAFLD: non-alcoholic fatty liver disease
NEAT1: nuclear enriched abundant transcript1
NF-κB: nuclear factor κB
NHL: non-Hodgkin lymphoma
NK: natural killer cells
NO: nitric oxide
NOX1: phagocyte-like NADPH oxidase 1
NPC: nasopharyngeal carcinoma
Nrf2: nuclear factor-erythroid related factor 2
OC: oral cancer
OGG1: 8-oxoguanine DNA glycosylase
OPG: osteoprotegerin
OR: odds ratio
P21: cyclin inhibition protein 2l
PAI-1: plasminogen activator inhibitor-1
p-AKT: phosphorylated protein kinase B
P-Akt: phospho-protein kinase B
PARP: poly ADP ribose polymerase
PC-12: pheochromocytoma cells
PCOS: polycystic ovary syndrome
PD-1: programmed death factor 1
PD-L1: programmed death factor ligand 1
PEF: pulsed electric field
PGE2: prostaglandin e2
P-gp: P-glycoprotein
PgR: progesterone receptor
PI3K: phosphatidylinositol 3-kinase
PIKK: phosphatidylinositol 3-kinase-related protein kinase
PIN1: peptidyl-prolyl isomerase NIMA-interacting 1
PKA: protein kinase A
PLCO: prostate, lung, colorectal and ovarian cancers
PMD: mammographic density
PP2A: protein phosphatase 2A
PPIase: peptidyl-prolyl *cis*-*trans* isomerase
Ptc: membrane receptors patched
RANK: receptor activator of nuclear factor κB
RARα1: retinoic acid receptor alpha 1
RKO: colorectal cancer cell-line
ROS: reactive oxygen species
RR: relative risk
SAPK: stress-activated protein kinase
SCC: squamous cell carcinoma
SEAHS: Southeastern Arizona Health Study
SFRP1: secreted frizzled-related protein 1
SHH: sonic hedgehog pathway
SHP1: Src homology containing protein tyrosine phosphatase 1
SKY: tyrosine protein kinase
Smad: drosophila mothers against decapentaplegic protein
Smo: membrane receptors smoothened
SN-38: 7-ethyl-10-hydroxycamptothecin
SOX2OT: sex-determining region y-box 2 overlapping transcript
SPHK1: sphingosine kinase 1
STAT3: signal transducers and activators of transcription
SUFU: suppressor of fused
SUM-149: breast cancer cell-line
SW780: bladder cancer cell-line
TGF-β1: transforming growth factor-β
THP-1: human monocytic leukemia cell-line
TIMPs: tissue inhibitor of matrix metalloproteinases
TLR-4: toll-like receptor 4
TME: tumor microenvironment
TMZ: temozolomide
TNBC: triple-negative breast cancer
TNF-α: tumor necrosis factor α
Tollip: toll interaction protein
TPA: 12-O-tetradecanoylphorbol-13-acetate
TRAIL: factor-related apoptosis-inducing ligand
TWIST 1: twist family bHLH transcription factor 1
uPA: urokinase plasminogen activator
VEGF: vascular endothelial growth factor
VEGFR2: vascular endothelial growth factor receptor 2
WAF1: wild-type p53 activated fragment 1
Wnt: wingless-type mice mammary tumor virus integration site family.
